# Supplementary material for: Expression of dlx genes in the normal and regenerating brain of adult zebrafish
Source: PLoS One. 2020 Jun 4;15(6):e0229549. doi: 10.1371/journal.pone.0229549 (PMC7272068; doi:10.1371/journal.pone.0229549)
Supplement: S1 Table — (DOC) [file pone.0229549.s001.doc]

| Gene of interest | Forward primer | Reverse primer | Fragment size | |
| --- | --- | --- | --- | --- |
| *dlx1a* | CAACTCGGTCGGTAGCCATT | GCTTGCGGATCTTTTTGCCT | | 176 bp |
| *dlx2a* | GAAACGCTTTCGGCCCCTA | CCATTCGGATTTCAGGTTCGC | | 96 bp |
| *dlx5a* | GGCTCATACTCCACAGCGTA | CATCCTTACTTCGGGCTCGG | | 105 bp |
| *dlx6a* | CAGCAGACTCAATACCTGGCA | TACCGCCTTGTTTCAACAGC | | 133 bp |
| *ef1a* | CTGGAGGCCAGCTCAAACAT | ATCAAGAAGAGTAGTACCGCTAGCATTAC | | 87 bp |
| *ywhaz* | TCTGCAATGATGTGTTGGAGC | TCAATGGTTGCTTTCTTGTCGTC | | 151 bp |
| *rpl13a* | TCTGGAGGACTGTAAGAGGTATGC | AGACGCACAATCTTGAGAGCAG | | 148 bp |
