# Supplementary figures and images for: Expression of dlx genes in the normal and regenerating brain of adult zebrafish
Source: PLoS One. 2020 Jun 4;15(6):e0229549. doi: 10.1371/journal.pone.0229549 (PMC7272068; doi:10.1371/journal.pone.0229549)

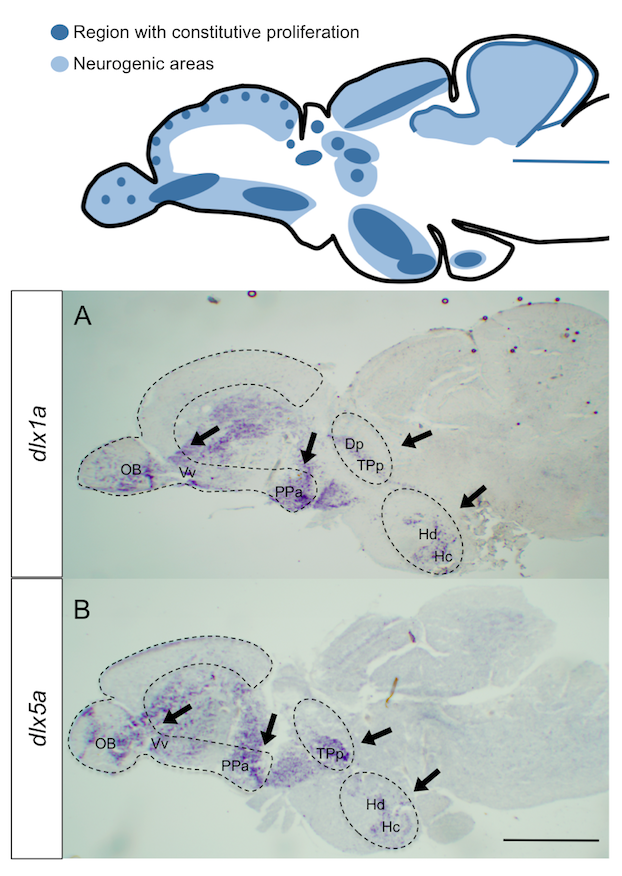

Supplement: S1 Fig — Upper panel shows drawing of an adult brain sagittal section depicting neurogenic areas and zones with constitutive proliferation (adapted from Kizil C. et al., 2011). [A-B] shows expression of dlx1a and dlx5a verified with ISH. Arrows indicate the main areas where expression of dlx genes matches areas with constitutive proliferation. These areas are: olfactory bulb (OB), ventral nucleus of ventral telencephalic area (Vv), parvocellular preoptic nucleus, anterior part (PPa), posterial zone of dorsal telencephalic area (Dp), periventricular nucleus of posterior tuberculum (TPp) and caudal zone of periventricular hypothalamus (Hc) and dorsal zone of periventricular hypothalamus (Hd). Scale bar: 1mm. (PNG) [file pone.0229549.s002.png]

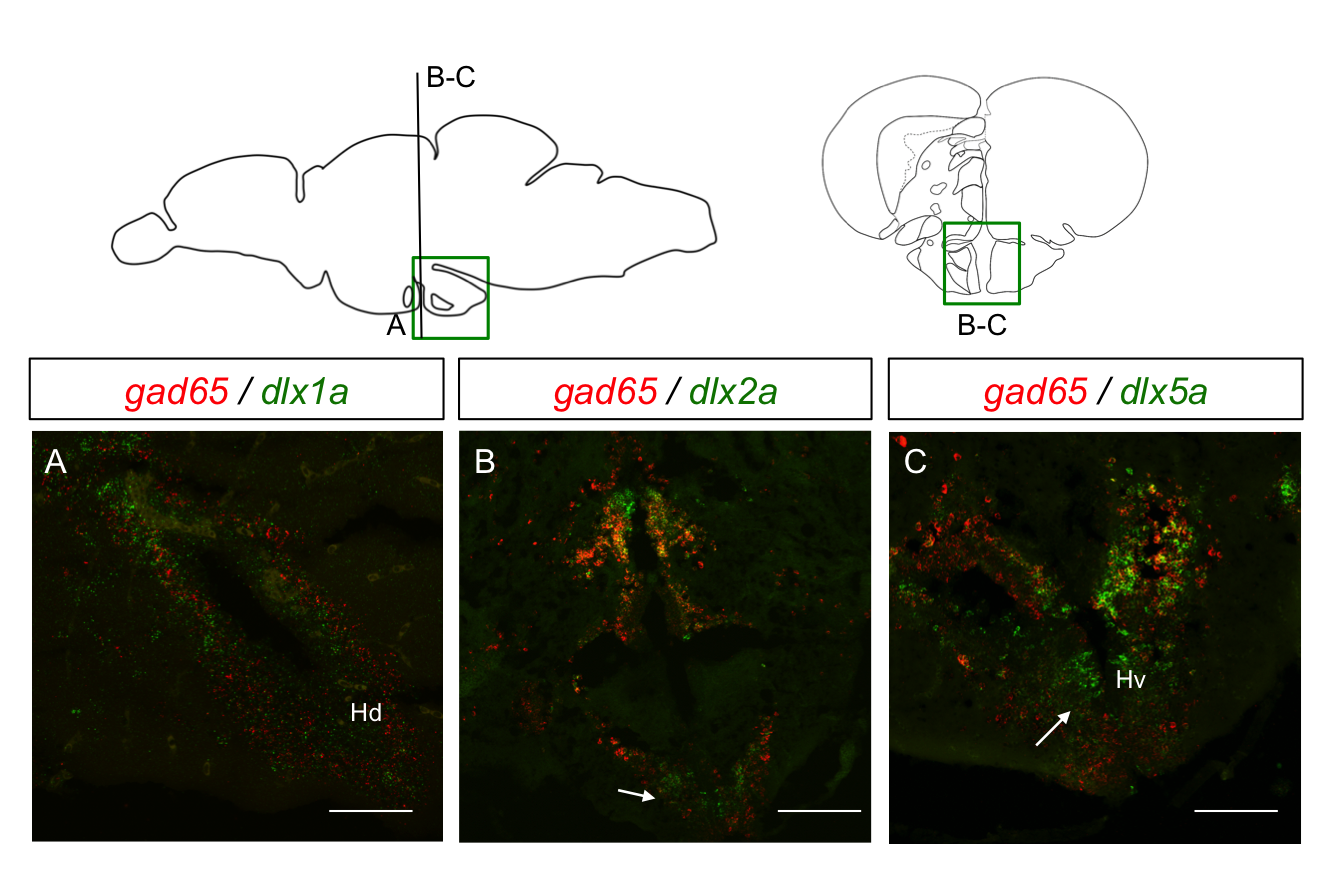

Supplement: S2 Fig — Double fluorescence in situ hybridization with transverse sections of the forebrain with (A-D) expression of dlx2a and dlx5a in green along with anatomical parts indicated and (E-H) expression of gad65 in red. (I-L) Co-localization of dlx and gad65 in yellow. Merged images were created with ImageJ(32) software. (N = 4 for dlx2a and dlx5a; N = 3 for dlx1a and dlx6a). Scale bar: 50μm. (PNG) [file pone.0229549.s003.png]

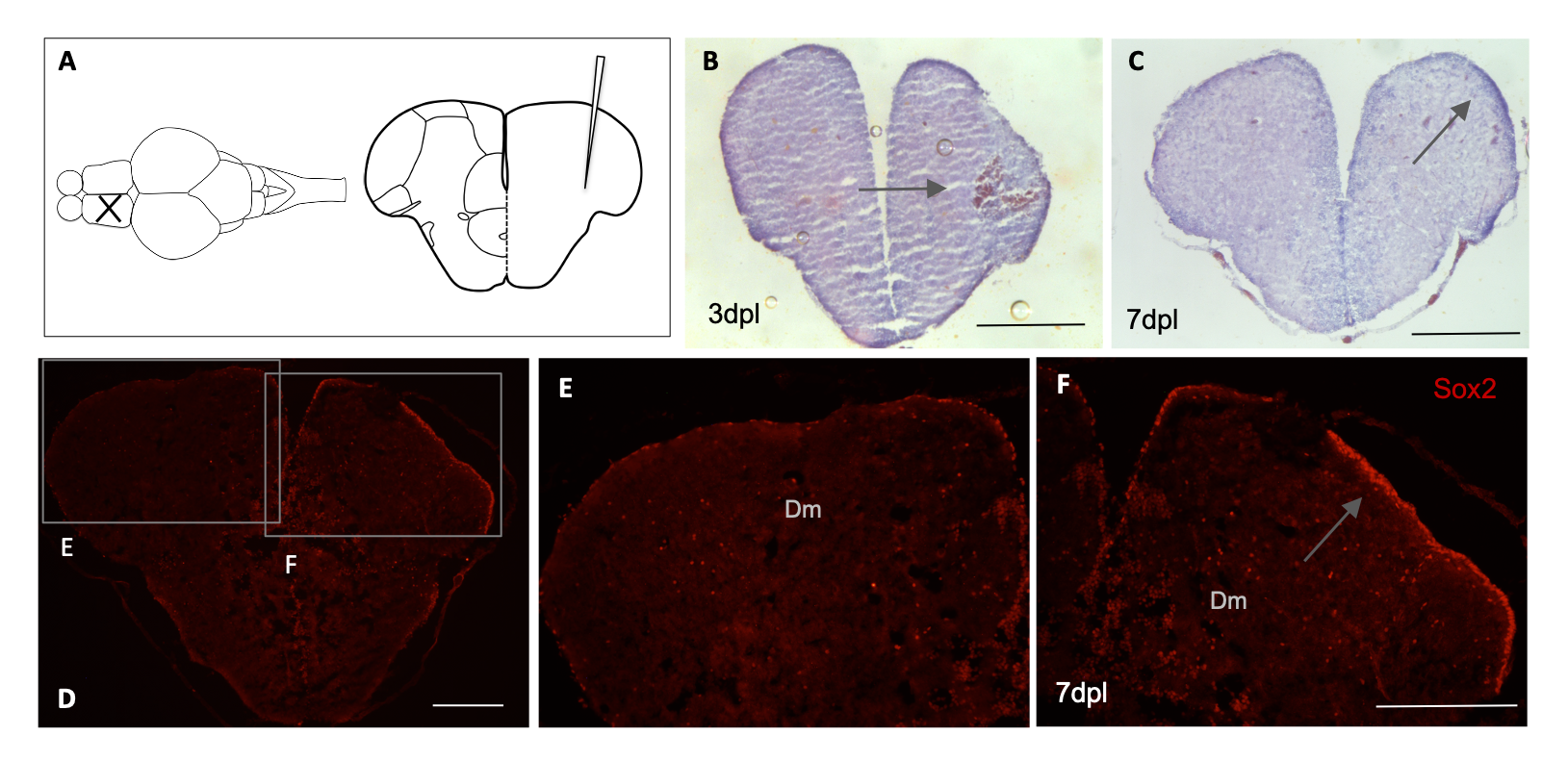

Supplement: S3 Fig — Schematic representation of lesion in the telencephalon area of adult zebrafish (A). Haematoxylin-Eosin staining and arrows indicate blood clot formation at 3 dpl (n = 3) (B) and apparent increase in cell number with higher intensity of staining at the lesioned hemisphere at 7dpl (n = 4) (C). Immunohistochemical labeling with Sox2 at 7 dpl indicates higher number of neural stem cells in the ventricular zone of the telencephalon (n = 3) (D). Magnification of image shown the contralateral hemisphere in E and regenerating hemisphere in F with increase in cells positive for Sox2 in the ventricular zone of the lesioned telencephalon. Scale bar (B-F): 200μM. (PNG) [file pone.0229549.s004.png]
